# Predict and Analyze Protein Glycation Sites with the mRMR and IFS Methods

**DOI:** 10.1155/2015/561547

**Published:** 2015-04-15

**Authors:** Yan Liu, Wenxiang Gu, Wenyi Zhang, Jianan Wang

**Affiliations:** ^1^College of Mathematics and Statistics, Northeast Normal University, 5268 Renmin Street, Changchun 130024, China; ^2^College of Computer Science and Information Technology, Northeast Normal University, 2555 Jingyue Street, Changchun 130117, China; ^3^Institute of Applied Mathematics and Intelligent Systems, Changchun Architecture & Civil Engineering College, Changchun 130607, China

## Abstract

Glycation is a nonenzymatic process in which proteins react with reducing sugar molecules. The identification of glycation sites in protein may provide guidelines to understand the biological function of protein glycation. In this study, we developed a computational method to predict protein glycation sites by using the support vector machine classifier. The experimental results showed that the prediction accuracy was 85.51% and an overall MCC was 0.70. Feature analysis indicated that the composition of *k*-spaced amino acid pairs feature contributed the most for glycation sites prediction.

## 1. Introduction

Glycation is one of the most important posttranslation modifications (PTMs) of proteins. Glycation is a two-step nonenzymatic reaction. First, generate the stable Amadori product based on the unstable Schiff base. Secondly, the advanced glycation end products (AGEs) are generated at the second step. According to the clinical researches [1], the advanced glycation end products are involved in a variety of human diseases, such as diabetes, Alzheimer's disease, and Parkinson's disease. The glycation mechanism might be a key to the treatment of the above diseases. Identification of the glycation sites in protein may provide guidelines to understand the biological function of proteins glycation.

It is important to note that glycation and glycosylation are different. Glycation is the result of typically covalent bonding of a protein or lipid molecule with a sugar molecule, such as fructose or glucose, without the controlling action of an enzyme. As opposed to the nonenzymatic chemical reaction of glycation, glycosylation is an enzyme-directed site-specific process. Five types of glycosylation are produced, including N-glycosylation, O-glycosylation, C-mannosylation, glypiation, and phosphoglycans linked through the phosphate of a phosphoserine [2].

Although some high-throughput proteomics experimental methods [3] have been developed to find posttranslational modification (PTM) sites [4–6], it is still difficult to confirm glycation sites by these methods. Several computational approaches to predict glycosylation sites have been reported. Li et al. [7] trained SVM based on physicochemical properties of amino acids and a 0/1 system which was only focusing on the O-glycosylation in mammalian proteins; Caragea et al. [8] used ensemble method to identify N-linked, O-linked, and C-linked glycosylation. Chen et al. [9] predicted mucin-type O-glycosylation serine/threonine (S/T) sites in mammalian proteins with the assistance of SVM based on the composition of *k*-spaced amino acid pairs (CKSAAP) encoding scheme.

Compared with the glycosylation, the determination of protein glycation sites was more difficult. Therefore, to the best of our knowledge, such computer methods for prediction of glycation sites were rarely mentioned in literatures except the GlyNN [10]. GlyNN was built by combining 60 artificial neural networks with a balloting procedure and obtained the maximal Matthews correlation coefficient (MCC) of 0.58 with the sequence size 23.

Here, we used support vector machine to develop a predictor for glycation sites of lysine. Amino acid occurrence frequency, amino acid factors, and the composition of *k*-spaced amino acid pairs (CKSAAP) were used to encode glycation site peptides. We reduced the support vector machine classifier input features dimension by utilizing the maximum relevance minimum redundancy (mRMR) method followed by the incremental feature selection (IFS) procedure. The experimental result showed that our predictor achieved an overall MCC of 0.7063. Feature analysis prompted that the CKSAAP encoding was efficient to capture a glycation site's characters. The detailed analysis results in this work may provide useful insights to detect glycation sites. A web server (PreGly) that implemented the proposed method was freely available: http://202.198.129.220:8080/GlycationPre/.

## 2. Materials and Methods

### 2.1. Dataset

In this study, we took the GlyNN [10] dataset as the benchmark dataset. It was convenient to compare the performance of PreGly and GlyNN, since they were built on the same dataset. Johansen et al. [10] collected experimentally validated glycation sites by searching hundreds of research papers manually. The whole dataset contained 89 glycation sites (positive samples) and 126 nonglycation sites (negative samples) from 20 proteins.

Subsequently, we extracted each glycation site peptide with the window size 23, with 11 residues upstream and 11 residues downstream of the glycation site. To make sure that each sequence window size was fixed to 23, we complemented a nonexisting residue “O” to the peptide, less than 23 amino acid residues. The window size fixed to 23 is rational and has been confirmed experimentally [10].

However, for the amino acid site near the end of the peptide, 23 may be a too much window size. We removed peptides which extended too many “O” residues to test the influence of “O” residues on the predictor result. The experimental result yielded a prediction accuracy of 82.74% (Sn = 71.84%, Sp = 0.9136, and MCC = 0.6527) and the prediction performance was also better than GlyNN [10]. To ensure the data integrity, we still applied the window size 23 on the entire data. The whole dataset was provided in the supporting information available online at http://dx.doi.org/10.1155/2015/561547 (Supporting Information S1).

### 2.2. Feature Space

#### 2.2.1. Amino Acid Occurrence Frequency Feature

We calculated the occurrence frequencies of the 20 native amino acids in given proteins [11]. Given a protein *R*, *L* is the length of the peptide in protein *R*, and the number of *i*th amino acid in the peptide is *n*_*i*_. We use *p*_*i*_ as the amino acid occurrence frequency feature:

(1) $$\begin{array}{c} p_{i}=\sqrt{\frac{n_{i}}{L}},\;\left(i=1,2,\ldots ,20\right). \end{array}$$

#### 2.2.2. **k**-Spaced Amino Acid Pairs Feature

The composition of *k*-spaced amino acid pairs (CKSAAP) encode scheme has been employed to predict various PTMs [6, 9]. Given 20 native amino acids and one complementary residue “O,” there are 441 basic amino acid pair types: AA, AC⁡,…, AW, AY, OO. The basic amino acid pair types are enlarged to the *k*-spaced amino acid pair types. For example, the space number *k* of “A^∧∧^A” is equal to 2. We examined three predictors built with the parameter *k* = 3,4, and 5 and obtained that the maximum accuracy was 85.51% with *k* = 4.

#### 2.2.3. Amino Acid Factors Feature

AA Index database [12] collected the various physicochemical and biochemical properties of amino acids. Atchley et al. [13] performed multivariate statistical analyses on AA Index to produce five multidimensional patterns of attribute covariation which reflected polarity (AA Factor 1), secondary structure (AA Factor 2), molecular volume (AA Factor 3), codon diversity (AA Factor 4), and electrostatic charge (AA Factor 5). These five factors have been successfully used to solve several different biology problems, such as [7, 11].

As mentioned above, we encoded each glycation site peptide by 21 features of amino acid occurrence frequency, 5 features of amino acid factors, and 441 × 4 features of *k*-spaced amino acid pairs. Therefore, for each peptide consisting of 23 amino acid residues, there were a total of 21 + 5 × 23 + 441 × 4 = 1900 features (Supporting Information S5).

### 2.3. The mRMR Method

The maximum relevancy minimum redundancy (mRMR) method [14] is used to rank features based on the criterion of maximum relevance to the target and the minimum redundancy between features. On the rank list, features with the small index are considered the “good” features, and these “good” features may provide more information for glycation site prediction.

The maximum relevancy criterion can be expressed as

(2) $$\begin{array}{c} \max I\left(\Omega_{t},c\right)=\frac{1}{\left|\Omega_{t}\right|}\sum_{f_{j}\in \Omega_{s}}I\left(f_{j},c\right), \end{array}$$

where *I*(*f*_*j*_, *c*) calculates the relevance between the feature *f* in *Ω*_*t*_ and the target *c*.

And the minimum redundancy (MR) can be represented as

(3) $$\begin{array}{c} \min R\left(\Omega_{s}\right)=\frac{1}{m}\sum_{f_{i}\in \Omega_{s}}I\left(f_{j},f_{i}\right),\;j=1,2,\ldots ,n, \end{array}$$

where *Ω*_*s*_ is the already-selected features set with the set size *m* and *Ω*_*t*_ is the to-be-selected features set with the set size *n*. The MR calculates the redundancy between the feature *f* in *Ω*_*t*_ and all the other features in *Ω*_*s*_.

Based on formulas (2) and (3), the mRMR method is

(4) $$\begin{array}{c} \underset{f_{j}\in \Omega_{t}}{\max}\left[I\left(f_{j},c\right)-\frac{1}{m}\sum_{f_{i}\in \Omega_{s}}I\left(f_{j},f_{i}\right)\right],\;\left(j=1,2,\ldots ,n\right). \end{array}$$

### 2.4. Incremental Feature Selection

Incremental feature selection (IFS) method [15] is used to select the optimal features. Features in the mRMR feature rank list are added one by one during the IFS procedure. Then, we construct *N* feature sets when there are *N* features on the list and the *i*th feature set is composed of *i* features.

### 2.5. Support Vector Machine

Vapnik [16] first proposed the support vector machine (SVM) algorithm. In principle, SVM is a two-class classifier. Given training vectors *x*_*i*_ ∈ *R* ^*n*^ and their class labels *y*_*i*_ ∈ (−1,1), *i* = 1,…, *N*, SVM solves the problem:

(5) min⁡ hhhl12ωT·ω+C∑i=1NξiSubject  to yi(ωT·xi+b)≥1−ξi, ξi≥0,

where *ω* is a normal vector perpendicular to the hyperplane and *ξ*_*i*_ are slake variables for allowing misclassifications.

The support vector machine has been widely used in bioinformation [9, 17]. In this work, we implement LIBSVM package [18] with RBF function. The two parameters penalty parameter *C* and kernel parameter *γ* are found by using a grid search strategy based on 10-fold cross-validation.

### 2.6. Performance Evaluation

In statistical prediction, three cross-validation methods are often used to examine a predictor for its anticipated accuracy: *K*-fold cross-validation test, independent dataset test, and jackknife test [3]. We chose the 10-fold cross-validation test to examine the quality of our predictor. During the 10-fold cross-validation test process, the peptide samples were divided into ten parts. Each part of them was in turn as test samples, and the remaining nine parts were as the train samples.

Four parameters, sensitivity (Sn), specificity (Sp), accuracy (Ac), and Mathew correlation coefficients (MCC), are used to evaluate the predictor performance:

(6) Sn=TPTP+FN,Sp=TNTN+FP,Ac=TP+TNTP+FP+TN+FN,MCC=TP×TN−FN×FPTP+FN×TN+FP×TP+FP×TN+FN,

where TP, TN, FP, and FN represent the true positive, the true negative, the false positive, and the false negative, respectively. MCC (Matthew correlation coefficient) reflects both the sensitivity and the specificity of a predictor.

## 3. Results and Discussion

### 3.1. The Predictors' Performance with Different **k**

In order to find the optimal *k* value of the CKSAAP feature encoding which can detect the glycation sites with high accuracy, we investigated the predictor performance of *k* = 3,4, and 5. The highest accuracy was 85.51% when *k* = 4 (Supporting Information S2).

### 3.2. The mRMR and IFS Results

**Figure 1:**
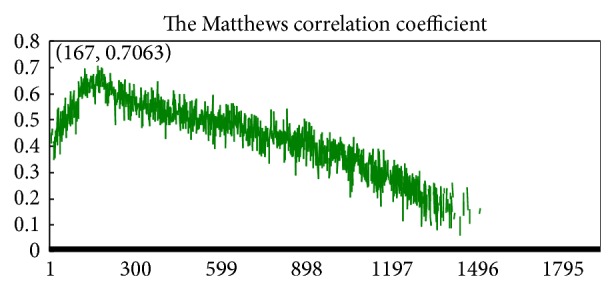
The MCC value against feature number.

First of all, by using the mRMR method, a total of 1900 features were ranked. Then we implemented the IFS procedure based on the mRMR rank list and generated 1900 feature sets. Subsequently, we built 1900 predictors and tested those predictors (Supporting Information S3) and plotted the IFS curve in Figure 1. It can be seen from Figure 1 that the maximum MCC was 0.7063 by using the top 167 features, and these 167 features (Supporting Information S4) were considered the optimal features to train our final predictor.

### 3.3. Optimal Features Analysis

In the 167 optimal features, over 90% features (153 CKSAAP features in 167 optimal features) were CKSAAP features. Thus, we inferred that the CKSAAP feature was the most importance feature for the prediction of glycation sites. We also listed the top 10 features of the optimal features in Table 1. It can be seen from Table 1 that there were 7 CKSAAP features in top 10 features. So we suggested that the CKSAAP feature was the most suitable feature for glycation sites prediction.

**Figure 2:**
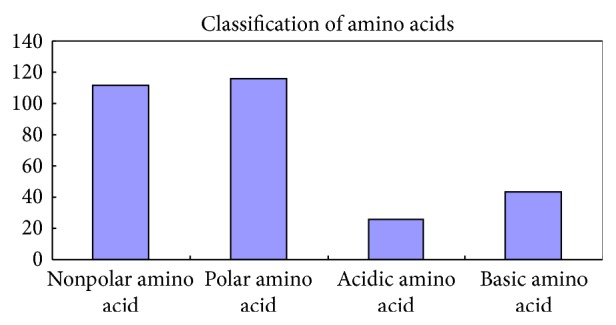
Amino acids classification distribution.

The feature selection methods could find out the most important CKSAAP pairs. For example, the first feature in Table 1 was the CKSAAP pair “S^∧∧∧∧^W,” which suggested that a potential glycation residue existed if the CKSAAP pair “S^∧∧∧∧^W” surrounding the residue have high abundance. We further investigated the classifications of the 20 native amino acids of the optimal features (Figure 2). From Figure 2, we can see that the polar amino acid and the nonpolar amino acid were vital characteristics for the glycation sites prediction.

**Figure 3:**
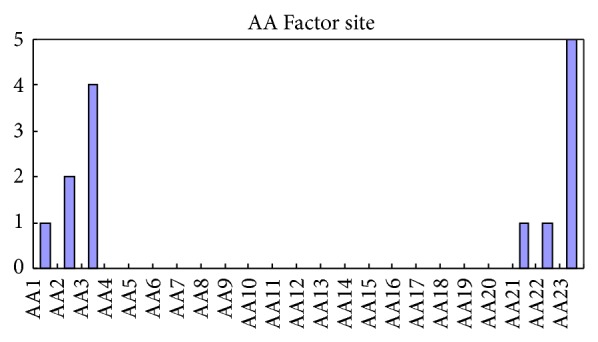
Number of corresponding specific sites.

We also noted that the AA Factor features played some roles in glycation sites' prediction. There were one polarity factor, two secondary structure factors, five molecular volume factors, one codon diversity factor, and five electrostatic charge factors in the 14 AA Factor features. And the sites distributions of AA Factor were shown in Figure 3. Figure 3 indicated that site 23 was the most important site for glycation sites' prediction and site 3 was the secondary important site for glycation sites prediction. Those results are consistent with the literature [10], which reported that the N-terminal parts of the human proteins have a higher predicted glycation potential than the other parts of the proteins.

### 3.4. Compare with Previous Method

In this section, we compared the proposed method with a previous method GlyNN [10]. As can be seen from Table 2, the performance of PreGly was better than that of GlyNN. The prediction accuracy of PreGly was 85.51% and the MCC was 0.70 with 167 optimal features. And from these better prediction results, we speculated that the CKSAAP encoding may provide more information than the consensus sequence motif.

### 3.5. The Performance of PreGly on Independent Dataset

We have reported that PreGly could achieve a better prediction performance. To objectively assess our predictor, we further tested our method on an independent dataset. 17 protein sequences containing experimentally validated glycation sites were retrieved from Uniport as the independent dataset (Supporting Information S6). Glycation sites labeled by “potential” and “probable” were removed. Finally, a total of 82 glycation sites and 117 not glycated sites in 17 proteins were retrieved. For each glycation site or not glycated site, a 23-residue peptide containing central glycation/not glycated site and 11 residues upstream and 11 residues downstream of the glycation/not glycated site was extracted. The peptides with length less than 23 were extended by “O” residue.

After tenfold cross-validation, the average prediction accuracy (Ac), sensitivity (Sn), specificity (Sp), and MCC of PreGly on the independent dataset were 79.92%, 64.2%, 91.57%, and 0.5849.

### 3.6. Discussion

**Figure 4:**
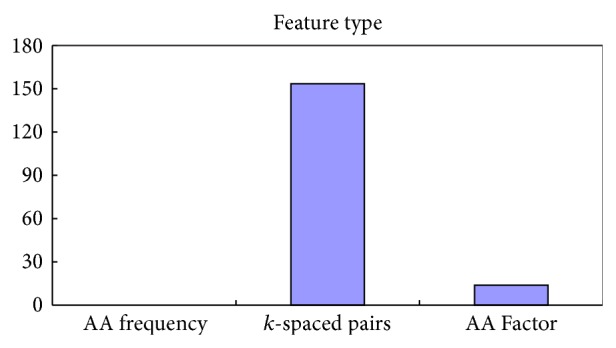
The distribution of each feature type in the optimal features set.

In the 167 optimal features, there were 153 CKSAAP features, 14 AA Factor features, and none of the amino acid occurrence frequency features. The feature distribution of optimal features set was shown in Figure 4. Considering the better performance of PreGly, it was possible that the CKSAAP [19] encoding was particularly suitable for the prediction of glycation site, which was to say that the short linear motifs may be more important than position-specific patterns in glycation sites' recognization. These analysis results reinforced the viewpoint that there may be no significant difference of mutations to other amino acids for each glycation site [20]. Figure 4 also implied that the amino acid occurrence frequency feature was faintly contributed to glycation sites prediction.

**Figure 5:**
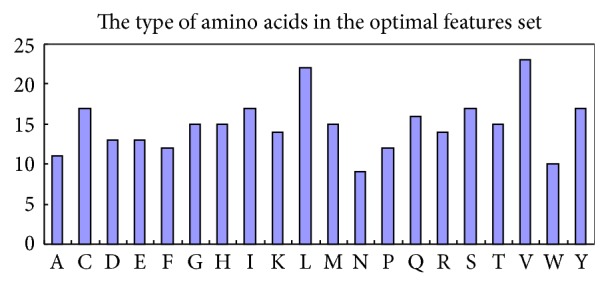
The distribution of the amino acid types in the optimal features.

The amino acids classification distribution (Figure 2) revealed that the polar amino acid and the nonpolar amino acid were effective for the glycation sites prediction. Apart from this, we further investigated the 20 native amino acids quantity in the optimal features (Figure 5). As shown in Figure 5, the numbers of Leucine and Valine were more than the other amino acids, whereas Asparagine and Tryptophan were the two kinds of amino acids in the least number. It was worthwhile to point out that the Asparagine and Tryptophan may provide useful clues to validate new glycation sites in protein sequences.

## 4. Conclusion

In this work, we built a predict model for protein glycation site prediction based on support vector machine. Our predictor reached an overall MCC of 0.706324, and sensitivity, specificity, and accuracy were 71.06%, 95.85%, and 85.51%, respectively. Glycation is involved in several diseases such as Alzheimer. It was advisable to identify glycation site for the associated diseases treatment. Detailed analysis conducted in this study may provide insights into understanding the mechanism of glycation and provide clues for the treatments of glycation related disease [21–28].

## Supplementary Material

The supplementary material includes the training dataset and the independent dataset of our manuscript.

## Figures and Tables

**Table 1:** Top 10 features of the optimal features.

Order	Feature number	Feature name	Explain
1	1793	S^∧∧∧∧^W	Pair of serine and tryptophan spaced with 4 residues
2	33	SS	The secondary structure of site 3
3	1494	C^∧∧∧∧^Q	Pair of cystine and glutamine spaced by 4 residues
4	1752	Q^∧∧∧∧^Y	Pair of glutamine and tyrosine spaced by 4 residues
5	91	EC	The electrostatic charge of site 14
6	710	H^∧∧^H	Pair of histidine and histidine spaced by 2 residues
7	129	MV	The molecular volume of site 22
8	341	L^∧^S	Pair of leucine and serine spaced by one residue
9	629	D^∧∧^L	Pair of aspartic acid and leucine spaced by 2 residues
10	210	E^∧^M	Pair of glutamic acid and methionine spaced by one residue

**Table 2:** Comparison of PreGly with GlyNN.

Method	Sensitivity (%)	Specificity (%)	Accuracy (%)	MCC
PreGly	71.06	95.85	85.51	0.70
GlyNN	78.65	80.15	79.50	0.58
